# Effect of Single Amino Acid Substitution Observed in Cancer on Pim-1 Kinase Thermodynamic Stability and Structure

**DOI:** 10.1371/journal.pone.0064824

**Published:** 2013-06-05

**Authors:** Clorinda Lori, Antonella Lantella, Alessandra Pasquo, Leila T. Alexander, Stefan Knapp, Roberta Chiaraluce, Valerio Consalvi

**Affiliations:** 1 Department of Biochemical Sciences “A. Rossi Fanelli”, Sapienza University of Rome, Rome, Italy; 2 UT-BIORAD-FARM CR Casaccia ENEA, Rome, Italy; 3 Nuffield Department of Clinical Medicine, University of Oxford, Oxford, United Kingdom; National Institute for Medical Research, Medical Research Council, London, United Kingdom

## Abstract

Pim-1 kinase, a serine/threonine protein kinase encoded by the *pim* proto-oncogene, is involved in several signalling pathways such as the regulation of cell cycle progression and apoptosis. Many cancer types show high expression levels of Pim kinases and particularly Pim-1 has been linked to the initiation and progression of the malignant phenotype. In several cancer tissues somatic Pim-1 mutants have been identified. These natural variants are nonsynonymous single nucleotide polymorphisms, variations of a single nucleotide occurring in the coding region and leading to amino acid substitutions. In this study we investigated the effect of amino acid substitution on the structural stability and on the activity of Pim-1 kinase. We expressed and purified some of the mutants of Pim-1 kinase that are expressed in cancer tissues and reported in the single nucleotide polymorphisms database. The point mutations in the variants significantly affect the conformation of the native state of Pim-1. All the mutants, expressed as soluble recombinant proteins, show a decreased thermal and thermodynamic stability and a lower activation energy values for kinase activity. The decreased stability accompanied by an increased flexibility suggests that Pim-1 variants may be involved in a wider network of protein interactions. All mutants bound ATP and ATP mimetic inhibitors with comparable IC50 values suggesting that the studied Pim-1 kinase mutants can be efficiently targeted with inhibitors developed for the wild type protein.

## Introduction

Pim-1 kinase belongs to a family of serine/threonine protein kinases (EC 2.7.11.1) that are encoded by the *pim* proto-oncogenes [1]–[3]. Pim-1 locus has been originally identified as a common Proviral insertion site in moloney murine leukemia virus-induced T-cell lymphomas in mice [4]. The encoded protein kinase is involved in several signalling pathways such as the regulation of cell cycle progression and apoptosis. The three Pim family members Pim-1, Pim-2 and Pim-3 identified in humans have been reported as signalling protein kinases playing an important role in tumor biology [5], [6]. In addition, many cancer types show high expression levels of Pim kinases. For instance Pim-1 and Pim-2 have been reported to be highly expressed in leukemia, lymphoma, prostate cancer and multiple myeloma and are considered to be involved in the initiation and progression of the malignant phenotype [7]. In addition, Pim-1 has been identified as a key cofactor regulating the expression of the oncogenic transcription factor c-Myc by phosphorylating serine 10 in histone H3 in enhancer region of the Myc locus and its target genes [8].

Expression of Pim-1 kinase can be induced by a variety of growth factors. Pim-1 homeostasis is important for normal cell function. Pim-1 activity is therefore tightly regulated at several levels including transcriptional, post-transcriptional, translational and post-translational. Several signal transduction pathways have been associated with the regulation of Pim-1 expression. For instance Pim-1 expression is stimulated by activation of the JAK/STAT pathway which also regulates its degradation via negative feedback loop. Pim-1 is overexpressed in many haematological malignancies and recent studies on Pim family kinases indicate that play important roles also outside of the hematopoietic system, as in the cells of several solid tumors [6]. Notably, in several cancer tissues somatic Pim-1 mutants have been identified [9]–[12]. Many of these variants are nonsynonymous single nucleotide polymorphisms (nsSNPs), single nucleotide variations occurring in the coding region and leading to a polypeptide sequence with amino acid substitutions [13]. Importantly, after growth factor stimulation Pim-1 protein levels are transient and the protein has a short half-life in cells being rapidly degraded by the ubiquitin system.

A number of investigations have addressed the effect of nsSNPs on protein stability, protein-protein interactions and protein functions [14]. At the same time, natural variants have been catalogued with the aim to distinguish between naturally occurring genetic differences, presumably with no functional consequences (passenger mutations) as those collected in the SNP database, and those associated with the development of diseases (driver mutations) [15], as the OMIM database [16], the Human Gene Mutation Database [17] and in particular those found to be associated with cancer (COSMIC) [11].

Computational analysis of identified mutants has predicted that around 30% of protein variants resulting from nsSNPs are less stable than the wild type variant [18] however, an experimental assessment of the stability of common variants is still needed to determine the effect of mutations on protein structure and function [19].

In this study we selected nsSNPs resulting in Pim-1 variants that are expressed in cancer tissues and reported in SNP database [9], [11], [20]. These Pim-1 variants have been comprehensively characterized to investigate the effect of single amino acid substitution on Pim-1 thermal and thermodynamic stability and structure in solution. Our study showed that all mutants studied lead to significant destabilization of Pim-1 kinase.

## Results

### Spectroscopic Characterization of Pim-1 Wild Type and Mutants Found in Cancer

**Figure 1:**
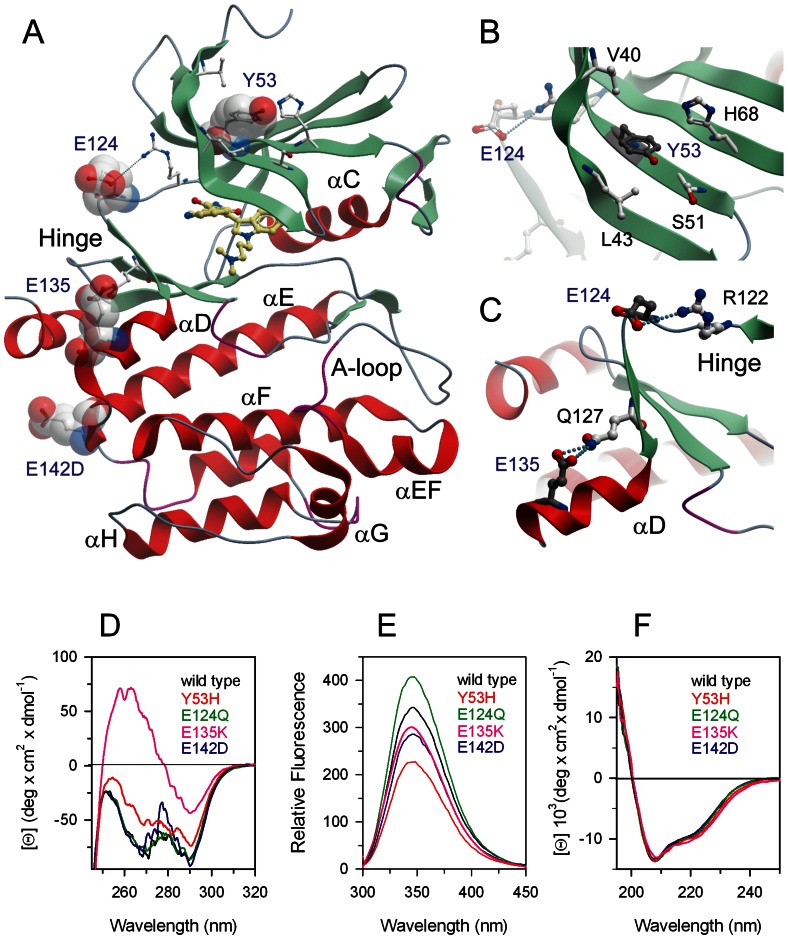
Location of Pim-1 mutations and spectral properties of wild type (black), Y53H (red), E124Q (green), E135K (pink) and E142D (blue). (**A**) Structure of Pim-1 (PDB code: 1XWS) shown as a ribbon diagram. Mutated residues are highlighted and the main secondary structure elements are shown. The ATP mimetic inhibitor cocrystallized in this structure is shown in ball and stick representation.(**B**) Detailed view of the local structural environment around the mutated residue Y53. (**C**) Detailed view of the local structural environment around the mutated residue E135. (**D**) Near-UV CD spectra were recorded in a 1.0-cm quartz cuvette at 1.4 mg/ml protein concentration in 20 mM Tris/HCl, pH 7.5 containing 0.2 M NaCl and 2 mM DTT. (**E**) Intrinsic fluorescence emission spectra were recorded at 0.04 mg/ml protein concentration (295 nm excitation wavelength) at 10°C in 20 mM Tris/HCl, pH 7.5 containing 0.2 M NaCl and 200 µM DTT. (**F**) Far-UV CD spectra were recorded in a 0.1-cm quartz cuvette at 0.2 mg/ml in 20 mM Tris/HCl, pH 7.5 containing 0.2 M NaCl and 0.4 mM DTT.

In this study we selected four mutations (Y53H, E124Q, E135K and E142D) that all have been detected in cancer for a detailed protein stability and structural analysis using spectroscopic techniques (Fig. 1). The mutants are located in the catalytic domain of Pim-1 kinase (Fig. 1A). Y53 is located in the N-terminal kinase lobe in the central β-sheet cradle and its amide nitrogen forms a hydrogen bond with I66 carbonyl group on beta-strand 3 (Fig. 1B). The residue E124 is located in the kinase hinge region and forms a salt bridge with R122 (Fig. 1C). The hinge region is a key element regulating the C-terminal lobe movement which may be affected by the E124Q mutation. E135 is located in helix αD forming a hydrogen bond with Q127 which is likely to be important in stabilizing this helix (Fig. 1C). Finally, E142 is a surface exposed residue and the conservative substitution with and aspartate is not likely to have a significant effect on protein stability, dynamics and structure.

The near-UV CD spectrum of wild type Pim-1 shows the spectral contribution of all aromatic residues and is characterized by two strong negative peaks centred at 290 nm and at 269 nm accompanied by fine structure features at 275–280 nm (Fig. 1D). The E124Q mutant displays a near-UV CD spectrum closely similar to that of the wild type except for a slight decrease of the fine structure at 275–280 nm. E142D displays a near-UV CD spectrum which surprisingly differs from the wild type spectrum in the 275–280 nm region. The near-UV CD spectrum of Y53H is dramatically different from the wild type spectrum and the other mutants: the fine structure at 275–280 nm is lost, the contribution at 290 nm is significantly decreased and the negative ellipticity at around 269 nm is markedly altered. E135K near-UV CD spectrum resembles that of Y53H with a markedly decreased dichroic activity in the 290–275 nm region and with a positive contribution in the region below 275 nm. The fluorescence emission spectra of wild type and mutants are all centred at the same maximum emission wavelength around 345 nm and show differences in emission fluorescence intensities (Fig. 1E). Far-UV CD spectra of all the mutants are virtually superimposable to that of Pim-1 wild type and typical of an alpha and beta protein, showing a local minimum at around 208 nm, a 200 nm zero intercept and a 1.52 ratio of the 208/222 bands (Fig. 1F).

### Temperature Dependence of Kinase Activity

**Figure 2:**
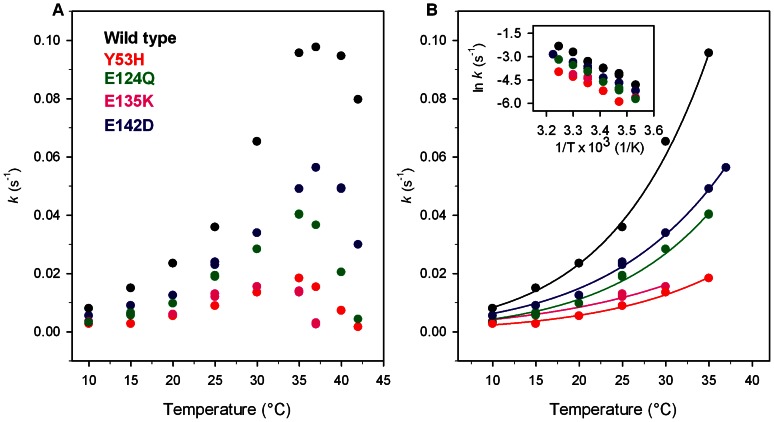
Effect of temperature on kinase activity of Pim-1 wild type (black), Y53H (red), E124Q (green), E135K (pink) and E142D (blue). (**A**) Temperature dependence of kinase activity of Pim-1 wild-type and mutants. (**B**) Non-linear fit of the temperature dependence of kinase activity to the Arrhenius equation (Eqn. 1); the inset shows the linear Arrhenius plot for the same data. Assays were performed under the conditions described in Materials and Methods, using 2.7 µM enzyme.

**Figure 3:**
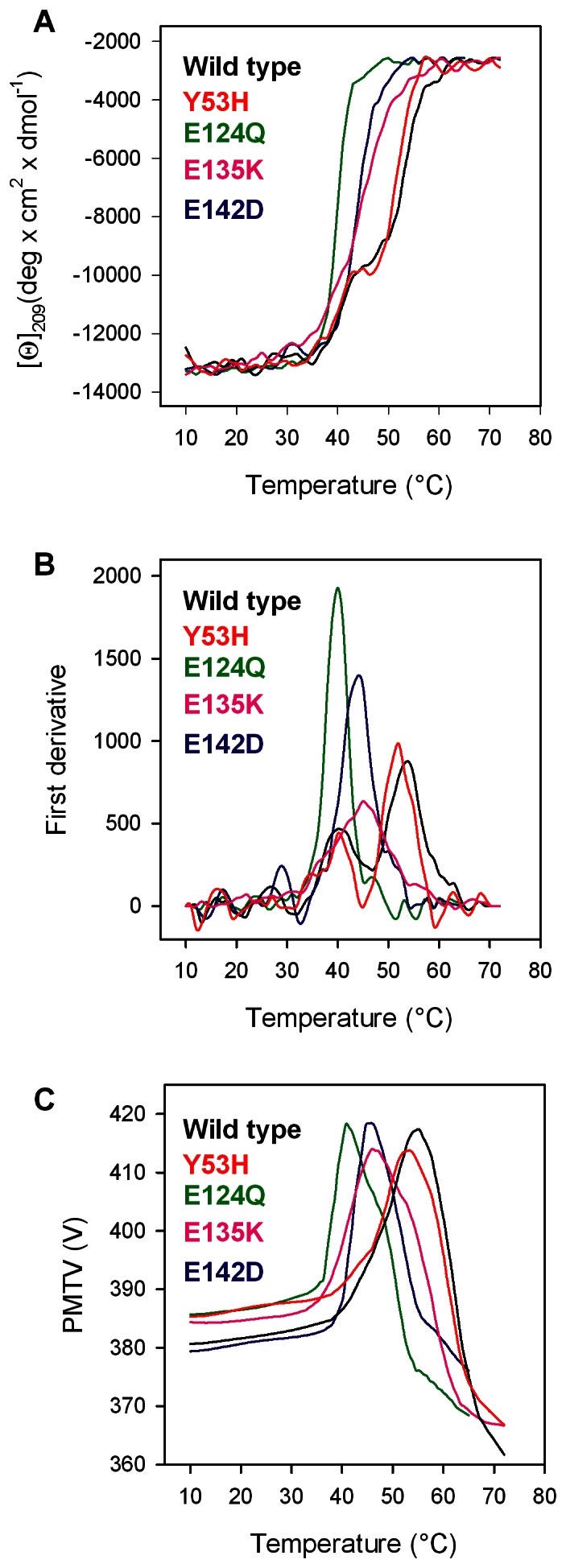
Thermal transition of Pim-1 wild type (black), Y53H (red), E124Q (green), E135K (pink) and E142D (blue). (**A**) Pim-1 wild type and mutants were heated from 10°C to 72°C in a 0.1-cm quartz cuvette at 0.2 mg/ml in 20 mM Tris/HCl, pH 7.5 containing 0.2 M NaCl and 0.4 mM DTT. The dichroic activity at 209 nm was monitored continuously every 0.5°C. (**B**) First derivative of the same data as in (**A**). (**C**) PMTV data recorded in the same experiments shown in (**A**).

The temperature dependence of kinase activity of Pim-1 wild type and its mutants was examined over the temperature range of 10–42°C (Fig. 2A). The optimal temperatures for catalysis, at constant ATP concentration, were estimated to be at 37°C for the wild type and E142D, at around 35°C for E124Q and Y53H, and at around 30°C for E135K (Table 1 and Fig. 2A). Notably, the kinase activity at 37°C of all Pim-1 mutants is significantly reduced and corresponds to 57, 37, 16 and 3% of that of the wild type protein for E142D, E124Q, Y53H and E135K, respectively. The activation energy, *E*_a_^‡^, determined by the Arrhenius equation (1) in the temperature range between 10°C and the optimal temperature of each protein is lower than that of the wild type (Table 1 and Fig. 2B). This result suggests an increased flexibility of all the variants compared to the wild-type, particularly evident for E135K. The comparison of temperature dependence of kinase activity with the structural thermal unfolding monitored at 209 nm (Fig. 3A) clearly indicates that all the variants are significantly less thermal resistant and more flexible than the wild type.

**Table 1:** Effect of temperature on kinase activity of Pim-1 wild type and mutants.

	Wild type	Y53H	E124Q	E135K	E142D
*E*_a_^‡^ (kcal/mol)	16.86±0.54	14.27±0.49	15.46±0.42	11.36±0.34	14.21±0.39
T_max_ (°C)	37	35	35	30	37
Kinase activity at T_max_ (s^−1^)	0.098	0.018	0.040	0.015	0.056

*E*_a_^‡^ was determined by the Arrhenius equation (Eqn. 1) in the temperature range between 10°C and the optimal temperature of each protein.

### Thermal Unfolding

The thermal stability of Y53H, E124Q, E135K and E142D was investigated by continuously monitoring the ellipticity changes at 209 nm in the temperature range between 10 and 72°C. Data of mutants were compared with that of the wild type protein (Fig. 3A). The parameter chosen to compare the transition curves of Pim-1 wild type and mutants is the melting temperature (T_m_) defined as the mid point of the denaturation process as calculated by plotting the first derivative of the molar ellipticity values as a function of temperature (Fig. 3B). The temperature-induced ellipticity changes at 209 nm, where the main amplitude was observed, occur in an apparent cooperative transition for E124Q and E142D, with T_m_ values of 40.0 for E124Q and 44.0°C for E142D. For E135K the transition appears less cooperative with a T_m_ value of 45.0°C and no detectable accumulation of unfolding intermediate(s), suggesting that the substitution of E135 with a lysine residue may induce a partial local unfolding and/or that this variant may exist as a heterogeneous population of folded states. For wild type and Y53H, the apparent three-state thermal transitions suggest the accumulation of unfolding intermediates with two T_m_ values. The first transition occurs with T_m_ values (T_m_1) at 40.5 and 40.0°C, the second (T_m_2) at 54.0, 52.0°C for wild type and Y53H, respectively (Table 2). The temperature-induced ellipticity changes for wild type and mutants are all coincident with the heat-induced increase of the photomultiplier tube voltage (PMTV) above 370 V (Fig. 3C), suggesting that the temperature-induced unfolding is accompanied by protein aggregation [21]. Aggregation occurred also when thermal scans were performed at a lower heating rate with a shift of the apparent T_m_ to lower temperatures; the differences between the apparent T_m_ of wild type and variants were the same as those measured at higher heating rate (data not shown). The observed transitions are irreversible, as indicated by the spectra measured at the end of the cooling phase that differ from those of the native proteins measured at the beginning of the thermal transitions. Furthermore, inspection of the cuvette at the end of the cooling phase revealed the presence of a large amount of precipitate in all the samples.

**Table 2:** Melting temperatures and thermodynamic parameters for urea-induced unfolding equilibrium of Pim-1 wild type and mutants measured by far-UV CD spectroscopy.

	Wildtype	Y53H	E124Q	E135K	E142D
T_m_1 (°C)	40.5	40.0	40.0	45.0	44.0
T_m_2 (°C)	54.0	52.0	–	–	–
Δ*G* ^H^_2_^O^(kcal/mol)	6.41±0.50	3.11±0.29	3.51±0.26	1.82±0.11	3.19±0.25
*m* (kcal/mol/M)	1.20±0.09	0.62±0.05	0.67±0.05	0.43±0.02	0.69±0.05
[Urea]_0.5_ (M)	5.34	5.01	5.28	4.23	4.65

The temperature-induced changes were followed by monitoring the ellipticity at 209 nm. The T_m_ values were calculated by taking the first derivative of the ellipticity at 209 nm with respect to temperature. T_m_1 and T_m_2 refer to the first and second transition observed for wild type and Y53H, respectively. For urea-induced unfolding equilibrium the data were obtained at 10°C in 20 mM Tris/HCl, pH 7.5, containing 0.2 M NaCl and 200 µM DTT by measuring circular dichroism ellipticity at 222 nm [Θ_222_]. Δ*G* ^H^_2_^O^ and *m* values were obtained from Eqn. 3; [Urea]_0.5_ was calculated from Eqn. 4. Data are reported as the mean ± SE of the fit.

### Effects of Pim-1 Mutations on ATP and Inhibitor Binding

Mutations identified in cancer are a frequent cause of drug resistance. We were therefore interested if inhibitor binding would be affected by the studied mutations. To address this we screened several known Pim-1 inhibitors of the beta-carboline and imidazopyridazole class [22], [23] by temperature shift assays [24]. Screening against a series of inhibitor derivatives revealed no significant differences in temperature shift values suggesting that all mutants bind these inhibitors with similar binding constant (Table S1 and Table S2). To confirm this we measured enzyme kinetic data on a subset of inhibitors. IC50 values of Pim-1 inhibitors K00487, K018444a and K00207a were 0.048, 0.010 and 0.007 mM, respectively, for Pim-1 wild type. These values were closely similar for the Pim-1 variants with the exception of the IC50 values for K00487 and K00207a that were 1.8- and 1.4-fold increased for Y53H. We concluded therefore that specific inhibitor binding is not significantly affected by the studied mutations.

### Urea-induced Equilibrium Unfolding Transitions

**Figure 4:**
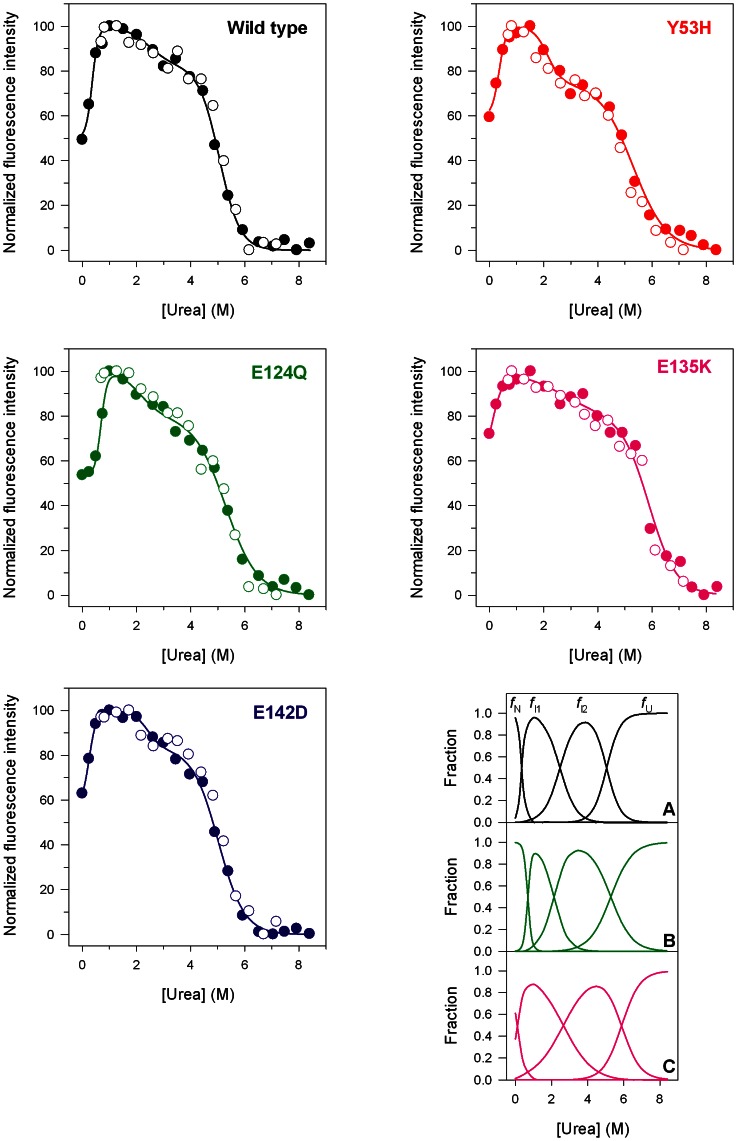
Urea-induced equilibrium unfolding of Pim-1 wild type (black), Y53H (red), E124Q (green), E135K (pink) and E142D (blue). Normalized relative fluorescence intensities at 345 nm; the continuous lines represent the nonlinear regression fit of the relative fluorescence intensities at 345 nm to Eqn. 5 calculated as described in Materials and Methods. The reversibility points are shown as empty circles and were not included in the nonlinear regression analysis. All spectra were recorded at 10°C as described in Materials and Methods. (**A–C**) The fraction of the native (*f*_N_), first intermediate (*f*_ I1_), second intermediate (*f*_I2_) and unfolded (*f*_U_) states, calculated as described in Materials and Methods using the thermodynamic parameters obtained from the fits of the equilibrium unfolding transitions to Eqn. 5, are shown for the wild type (**A**), E124Q (green) (**B**) and E135K (pink) (**C**).

**Figure 5:**
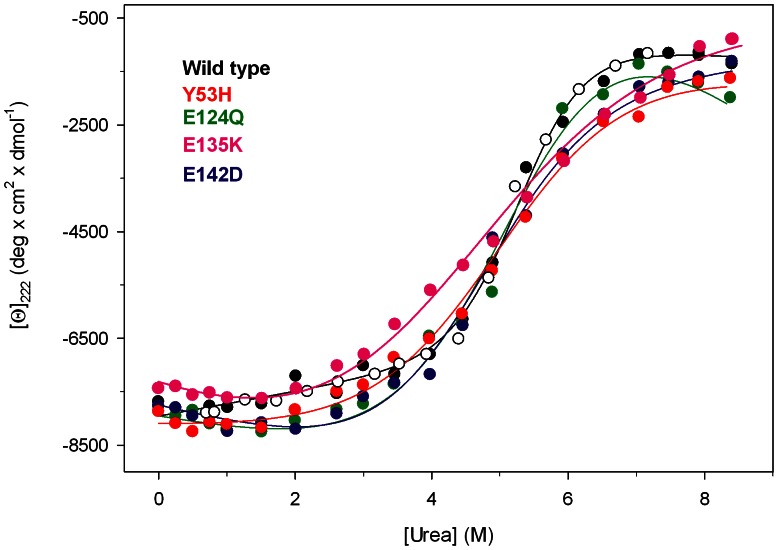
Urea-induced equilibrium unfolding of Pim-1 wild type (black), Y53H (red), E124Q (green), E135K (pink) and E142D (blue). Molar ellipticity at 222 nm ([Θ]_222_) reported after removal of the high-frequency noise and the low-frequency random error by SVD. Continuous lines are the nonlinear regression to Eqn. 3 of the data at varying denaturant concentrations, as described in Materials and Methods. The reversibility points (empty symbols) are shown, for clarity, only for the wild type and were not included in the nonlinear regression analysis. All spectra were recorded at 10°C as described in Materials and Methods.

Pim-1 wild type and variants reversibly unfold in urea at 10°C in 20 mM TrisHCl, pH 7.5, containing 200 µM dithiothreitol (DTT) and 0.2 M NaCl. The effect of increasing urea concentrations (0–8 M) on the structure of Pim-1 mutants was analyzed by far-UV CD and fluorescence spectroscopy and compared to the effect exerted on the wild type. The intrinsic fluorescence emission intensity at 345 nm of Pim-1 wild type and variants changes after 30 min incubation at 10°C, a time sufficient to reach equilibrium (Fig. 4). The plot of the relative fluorescence intensity changes versus increasing denaturant concentration shows complex profiles for the wild type and all the mutants (Fig. 4), suggesting a non two-state unfolding process and the population of denaturation intermediate(s). The complexity of the unfolding profiles may be ascribed to the multi-domain architecture of Pim-1 containing tryptophan residues in both the N-terminal and C-terminal lobes of the protein. At the end of the transition, above 7 M urea, the intrinsic fluorescence emission intensity at 345 nm is decreased about 1.3 fold (Fig. 4) and the maximal fluorescence emission wavelength shifts to around 357 nm either for the wild type and all the variants (data not shown). The same samples used to monitor the fluorescence emission changes (Fig. 4) during the unfolding transition were used to monitor far-UV circular dichroism ellipticity (Fig. 5) to allow a direct comparison with the fluorescence data. The urea-induced changes in 222 nm ellipticity of all the mutants are similar to that of the wild type, show a sigmoidal dependence on urea concentration and follow an apparent two-state transition without any detectable intermediate (Fig. 5). The unfolding process is fully reversible upon dilution of the denaturant either for the wild type or for all the mutants (Fig. 5) with transition midpoints between 4.23 and 5.34 M urea (Table 2). Table 2 shows the thermodynamic parameters values obtained for wild type and mutant forms of Pim-1 from the far-UV CD transition data. Pim-1 wild type is significantly more stable than all the variants, as indicated by comparison of Δ*G* values that in the case of E135K is about 3.5-fold lower than that of the wild type (Table 2). The decrease in Δ*G* may be mainly referred to the lower *m* values observed for all the variants with respect to the wild type. These results indicate that the change in the solvent exposed surface area upon unfolding is smaller for all the Pim-1 variants than for the wild type protein. Notably, the *m* values determined for Pim-1 wild type and its variants (Table 2) are five-fold lower than that predicted for a monomeric protein of 272 amino acid residues unfolded in urea [25]. The low values of *m* may be related to multi-state equilibrium unfolding, in line with the results obtained monitoring the unfolding process by intrinsic fluorescence emission intensity (Fig. 4).

The urea-induced unfolding transitions monitored by relative fluorescence intensity changes (Fig. 4) were analyzed using a four-state transition model to equation 5 as reported in Materials and Methods. The thermodynamic parameters relative to the unfolding process are reported in Table 3. The data indicate that the first transition from the native state (N) to the first intermediate (I_1_) occurs for Pim-1 wild type and all the mutants below 1 M urea with a larger Δ*G* value for E124Q and a lower Δ*G* value for E135K. The second transition from I_1_ to the second intermediate (I_2_) occurs in a very similar urea concentration range for all the Pim-1 mutants examined and the Δ*G* values for E142D and Y53H suggest a stabilization of the intermediate for these two variants (Table 3). The Δ*G* value relative to the last transition to the denatured state (U) is significantly larger for the wild type compared to the variants. In line with the results obtained from far-UV CD sigmoidal transitions (Table 2), the sum of the Δ*G* values for the three transitions is larger for the wild type, compared to the other mutants, with the exception of E142D whose Δ*G*_tot_ is similar to that of the wild type. Notably the Δ*G*_tot_ of E135K is significantly lower than that of all the other variants. The discrepancy between the thermodynamic parameters obtained by far-UV CD and fluorescence intensity strongly supports the presence of unfolding intermediates. The lack of a detectable intermediate by far-UV CD may indicate that the intermediate states detected by fluorescence represent conformational changes which occur in the proximity of any of the tryptophan residues, with alternative tertiary arrangements. It is well established that fluorescence intensity is extremely sensitive to the environment of a fluorophore and is considered as one the most direct signals that can be used to monitor thermodynamics of unfolding transitions [26]. To evaluate the fractional accumulation of the different species upon urea-induced unfolding, the equilibrium distribution of the four species, N, I_1_, I_2_ and U as a function of denaturant concentration could be reconstituted using the fitted values of Δ*G*_1_, Δ*G*_2_, Δ*G*_3_, *m*_1_, *m*_2_ and *m*_3_ reported in Table 3. The equilibrium distribution of N, I_1_, I_2_ and U is similar for wild type (Fig. 4A) and all the variants, however, some differences can be observed (Fig. 4B–C). The fraction of N (*f*_N_) is increased for E124Q (Fig. 4B) and significantly smaller for E135K (Fig. 4C), and similar to that of the wild-type (Fig. 4A) for all the other Pim-1 variants (data not shown). The fraction of I_1_ (*f*_I1_), which accumulates at 1.0 M urea for all the species, is slightly larger for E135K and smaller for E124Q. The fractional distribution of the second denaturation intermediate I_2_ (*f*_ I2_), which accumulates at about 4.0 M urea, appears modestly increased for Y53H, E124Q and E135K, compared to the wild type (data not shown). Notably, for the wild type and all the variants the unfolding intermediates I_1_ show the same maximal emission wavelength of N centred at about 345 nm, whereas that of I_2_ is shifted to around 348 nm. The detailed analysis of Pim-1 unfolding transitions suggests that significant differences in domain plasticity and unfolding occur in Pim-1 mutants when compared with the wild type protein.

**Table 3:** Thermodynamic parameters for urea-induced unfolding equilibrium of Pim-1 wild-type and mutants.

	Wild type	Y53H	E124Q	E135K	E142D
Δ*G*_1_ (kcal/mol)	1.83±0.11	1.54±0.15	3.46±0.24	0.28±0.04	1.03±0.11
*m*_1_ (kcal/mol/M)	5.07±0.30	4.39±0.44	5.03±0.35	2.69±0.37	4.17±0.46
[Urea]_0.5_ (M)	0.36	0.35	0.69	0.10	0.25
Δ*G*_2_ (kcal/mol)	3.21±0.38	4.75±0.57	3.08±0.34	1.94±0.23	5.22±0.68
*m*_2_ (kcal/mol/M)	1.30±0.15	2.23±0.27	1.45±0.16	0.73±0.09	2.18±0.28
[Urea]_0.5_ (M)	2.47	2.13	2.12	2.66	2.39
Δ*G*_3_ (kcal/mol)	7.71±0.46	4.97±0.40	5.22±0.36	6.64±0.46	6.12±0.49
*m*_3_ (kcal/mol/M)	1.53±0.09	0.94±0.07	0.99±0.07	1.13±0.08	1.22±0.09
[Urea]_0.5_ (M)	5.04	5.29	5.27	5.88	5.02
Δ*G*_tot_ (kcal/mol)	12.75	11.26	11.76	8.86	12.37

Urea-induced unfolding equilibrium data were obtained at 10°C in 20 mM Tris/HCl, pH 7.5, containing 0.2 M NaCl and 200 µM DTT by measuring the relative fluorescence intensity at 345 nm. The free energy of unfolding from the native state to the intermediate 1 (Δ*G*_1_), from the intermediate 1 to the intermediate 2 (Δ*G*_2_) and to the unfolded state (Δ*G*_3_) were calculated from Eqn. 5 **.** [Urea]_0.5_ and *m* which are the midpoint and *m* value for the transition between native, intermediate and unfolded states, respectively, were calculated from Eqn.5. Δ*G*_tot_ is obtained as the sum of Δ*G*_1,_ Δ*G*_2_, and Δ*G*_3._ Data are reported ± SE of the fit.

## Discussion

This study represents, to our knowledge, the first spectroscopic and thermodynamic characterization of human kinase Pim-1 and some of its disease relevant mutants found in cancer. We investigated the effect of amino acid substitution on the thermal and thermodynamic stability of wild type Pim-1 and compared these results with four Pim-1 variants, Y53H, E124Q, E135K and E142D, reported in SNPs database [9]–[12], [15], [20]. Examination of the Pim-1 crystal structure showed that most mutations are located close to structural elements important for kinase function and that form polar interactions with neighbouring residues.

The point mutations in the variants significantly affect the conformation of the native state of Pim-1. The differences in near-UV CD between mutants and wild type suggest that the tertiary contacts are significantly altered for all the mutants and that the single aminoacid substitution affects Pim-1 tertiary structure and lead to profound changes in the overall protein tertiary fold. In particular, the substitution of Y53 with histidine leads to dramatic tertiary structure changes, as revealed from the loss of the fine structure at 260–280 nm (Fig. 1D). The N-terminal lobe mutant Y53H displays the most significant changes in tertiary contacts, as judged on the basis of near-UV CD spectrum. Notably, the near-UV CD spectrum of the E124Q mutant appears more similar to that of the wild type, probably because the change of a charged polar Glu residue into the uncharged polar Gln residue has only a minor effect on the tertiary structure and its stability.

The Tyr53 residue is placed in the middle of beta-strand 2 and its amide nitrogen is hydrogen-bonded to the I66 carbonyl on beta-strand 3. In Y53H, the substitution of Y53 with a polar histidine may lead to a new hydrogen bond with H68 (Fig. 1). In addition, the proximity of Y53 to phosphate-binding P-loop, a region that plays an important role in the specificity and affinity of inhibitors [27], suggests that mutation of this residue may influence inhibitor binding.

The other mutation found in cancer, E124Q, concerns a residue located in the hinge region (121–126). The OE2 of E124 forms a salt bridge with the H11 of the neighboring R122 (Fig. 1). The substitution of E124 for glutamine (E124Q) destroys the salt bridge in the hinge region that may determine mobility of the N-terminal (33–121) and C-terminal (128–305) lobes. Notably, since the lobes often rotate or close around the hinge upon inhibitors binding the mutation of E124 significantly alters the dynamic properties of the Pim-1 and may change inhibitors affinity. Interestingly, this glutamate-arginine salt bridge is not present in the Pim-2 isoform possibly contributing to the lower stability of this protein [28].

The reversibility of the urea induced equilibrium unfolding at low temperature allows a quantitative determination of the effect of mutations on the thermodynamics of Pim-1 unfolding. All the mutants, expressed as soluble recombinant proteins, show a decreased thermal and thermodynamic stability (Table 2 and 3). The destabilizing effect of all the amino acid substitutions is particularly evident from the decrease in melting temperature monitored by secondary structure changes that suggests a higher flexibility of the variants with respect to the wild type. The decrease in melting temperature of all the Pim-1 variants studied is paralleled by a significant decrease in the Δ*G* ^H^_2_^O^ relative to the urea-induced unfolding compared to the wild type. These results are surprising since all the mutants are less stable than the wild type and Pim-1 is a proto-oncogene, thus one may conclude that Pim-1 variants are less efficient as oncogenes than the wild type. However, the decreased stability is accompanied by an increased flexibility, as indicated by the lower activation energy values for kinase activity observed in all the variants in comparison to that of the wild type, suggesting that Pim-1 variants may be involved in a wider network of protein interactions.

Notably, the transition midpoints for the spectral changes of all the mutants are not significantly changed with respect to the wild type; hence, the decrease of Δ*G* ^H^_2_^O^ values is mainly due to a decrease in *m* values. Thus the effect of the amino acid substitution on Pim-1 stability can be mainly referred to a decrease in the change of the solvent exposed surface area upon unfolding for all Pim-1 variants.

In conclusion our results indicate that the effect of the mutation observed in cancer tissues are directed to local changes of tertiary structure that however do not affect binding to type I kinase inhibitors studied.

## Materials and Methods

### Site-directed Mutagenesis

Pim-1 wild type enzyme plasmid was obtained by SGC (Oxford). Quick Change Site-Directed Mutagenesis Kit (Stratagene) was used to introduce the single mutations on wild type Pim1 plasmid used as template. The mutagenic oligonucleotides used are listed in Table 4.

**Table 4:** List of oligonucleotides used for site-directed mutagenesis.

Mutant	Primer sequences (5′ to 3′)
Y53HFW	GCTTCGGCTCGGTCCACTCAGGCATCCG
Y53HREV	CGGATGCCTGAGTGGACCGAGCCGAAGC
E124Q FW	CCTGATCCTGGAGAGGCCCCAGCCGGTGC
E124Q REV	GCACCGGCTGGGGCCTCTCCAGGATCAGG
E142D FW	GCCCTGCAAGAGGATCTGGCCCGCAGC
E142D REV	GCTGCGGGCCAGATCCTCTTGCAGGGC
E135 KFW	CGACTTCATCACGAAAAGGGGAGCCCTGCAAGAGGAGC
E135 REV	GCTCCTCTTGCAGGGCTCCCCTTTTCGTGATGAAGTCG

### Protein Expression and Purification

Recombinant Pim-1 protein has been expressed and purified as described in [29] with minor modifications. Pim-1**wild type and mutants were expressed in *E. coli* strain BL21(DE3). 10 ml of overnight culture was grown at 37°C in 1 l LB media containing ampicillin as antibiotic at a final concentration of 50 µg/ml until optical density OD_600_ reached 0.6. The culture was cooled on ice for 20 min, then the protein expression was induced overnight by adding 0.5 mM isopropyl-β-D-thiogalactoside (Sigma-Aldrich) and grown overnight at 15°C with energic shaking. The culture was harvested by centrifugation and resuspended in 50 ml of Binding buffer (50 mM Hepes, 500 mM NaCl, 5 mM Imidazole, 5% Glycerol, pH 7.5) containing 0.5 mM *tris*(2-carboxyethyl)phosphine (TCEP), and stored at −20°C until use. The cells were thawed on ice supplemented with protease inhibitors (Complete, Roche) and disrupted by sonication. The lysate was cleared by centrifugation and the supernatant was loaded on a DE52 column (GE Healthcare), previously equilibrated with Binding buffer and 0.5 mM TCEP, to remove nucleic acids. The flow-through was loaded on a Ni-NTA (Ni^2+^- nitriltriacetate) affinity column (GE Healthcare) pre-equilibrated with Binding buffer. The column was washed with Binding buffer to elute weakly bound contaminants. The recombinant protein was eluted by passing over the column binding buffer solutions containing increasing imidazole concentrations (50 mM, 100 mM, 150 mM and 250 mM, respectively). The collected eluates were supplemented with a final concentration of 10 mM DTT and tested for purity on SDS gel using precasted gel system (Invitrogen). The pure fractions were incubated overnight with tobacco etch virus protease (Pro-TEV), to remove the hexahistidine tag. After digestion, the protein was concentrated to 2 ml using Millipore concentrators and loaded onto a Superdex 200 300/10 gel filtration column on AKTA FPLC system previously equilibrated with 50 mM Tris/HCl, 0.25 M NaCl, 10 mM DTT, pH 7.5 at a flow rate of 1.0 ml/min. 2 ml fractions were collected and the pure protein was identified by SDS PAGE. Protein concentration was determined spectrophotometrically using a molar absorptivity of 48930 M*^−^* ^1^ cm^−1^at 280 nm based on a molecular mass of 35.685 kDa.

### Spectroscopic Measurements

Intrinsic fluorescence emission measurements were carried out at 10°C with a LS50B spectrofluorimeter (Perkin-Elmer), at 40.0 µg/ml protein concentration, using a 1.0 cm path length quartz cuvette. Fluorescence emission spectra were recorded from 300–450 nm (1 nm sampling interval), with the excitation wavelength set at 295 nm. Far-UV (190–250 nm) CD spectra were recorded either at a protein concentration of 0.20 mg/ml (0.4 mM DTT) in a 0.1 cm cuvette or at 40.0 µg/ml (0.2 mM DTT) in a 0.5 cm cuvette; near-UV (250–320 nm) CD spectra were recorded at a protein concentration of 1.40 mg/ml (2.0 mM DTT) in a 1.0 cm cuvette. Far- (190–250 nm) and near-UV (250–310 nm) CD spectra were measured using 0.1, 0.5 and 1.0 cm path length quartz cuvettes and the results obtained were expressed as the mean residue ellipticity (Θ), assuming a mean residue molecular mass of 110 per amino acid residue. All spectroscopic measurements were carried out at 10°C in 20 mM Tris/HCl, pH 7.5 containing 0.2 M NaCl.

### Temperature Dependence of Kinase Activity

The activity assay mixture in 0.5 ml final volume was incubated at increasing temperature in a thermostated cuvette. Reaction was started by adding 10–40 µl of purified enzyme at 10°C to 0.5 ml assay mixture equilibrated at the desired temperature. The final enzyme concentration was 2.7 µM. The solution was thoroughly mixed by pipetting and the absorbance at 340 nm was continuously monitored for 20 min in the thermostated cuvette. The temperature dependence was fitted nonlinearly to the Arrhenius equation using GraphPad Prism 4.0 to obtain the activation energies (*E*_a_^‡^) for the catalytic reaction
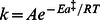
(1)where *k* (s^−1^) is the rate constant at temperature *T* (K), *A* is a reaction specific quantity, *R* the gas constant (1.987 cal×mol^−1^×K^−1^) and *E*_a_^‡^ the activation energy of the reaction. Hence, a plot of ln *k* versus 1/*T* gives a straight line with the slope being − *E*_a_^‡^/*R*.

### Kinase Assay

Phosphorylation reaction was monitored using a coupled-enzyme assay in which ADP production is coupled to NADH oxidation by pyruvate kinase and lactate dehydrogenase. The assay was carried out in a total volume of 0.5 ml in 50 mM HEPES, pH 7.5 containing 150 mM NaCl, 10 mM KCl, 2 mM DTT, 10 mM MgCl_2_, 1.0 mM phosphoenolpyruvate, 0.2 mM NADH, 15 U/ml pyruvate kinase, 30 U/ml lactate dehydrogenase, and 12.5 µM consensus peptide (AFRREWSPGKEAKK) as substrate. The reaction was monitored at 340 nm on a λ16 spectrophotometer (Perkin-Elmer) in the temperature range between 10 and 42°C, using a Peltier thermocontroller. After a 5 min preincubation of the reaction mixture at the assay temperature, the reaction was started by addition of 0.1 mM ATP a concentration well below the *K*_m_ value of 205.2 µM measured for the wild type protein. In the Caliper mobility shift assays kinase reactions were analyzed by microfluidic capillary electrophoresis. The final assay buffer was 50 mM HEPES, pH 7.5, 1 mM DTT, 0.02% Tween 20, 0.02% BSA, 10 mM beta-glycerophosphate, 0.01 mM Na_3_VO_4_, 0.6% DMSO, 8 mM MgCl_2_, 2 µM peptide and 0.2 nM kinase. The reactions were started by addition of substrate mix consisting of ATP and peptide substrate to kinase and inhibitor solution. ATP concentrations were adjusted to the K_m_values of the specific Pim enzyme. After incubation for 60 min at 30°C reactions were terminated with 100 mM HEPES pH 7.5, 5% DMSO, 0.1% coating reagent (Caliper Lifescience) 10 mM EDTA pH 8.0, 0.015% Brij35. Levels of substrate and product were quantified by measuring the laser-induced fluorescence intensities of fluorescein labels of the peptide. After turnover calculations, the dose-dependent response of the compound on kinase activity was established and expressed as IC50.

### Inhibitor Binding by Temperature Shift Assays

Thermal melting experiments were carried out using an Mx3005p Real Time PCR machine (Stratagene). Proteins were buffered in 10 mM HEPES pH 7.5, 500 mM NaCl and assayed in a 96-well plate at a final concentration of 2 µM in 20 µl volume. Compounds were added at a final concentration of 10 µM. SYPRO Orange (Molecular Probes) was added as a fluorescence probe at a dilution of 1∶1000. Excitation and emission filters for the SYPRO-Orange dye were set to 465 nm and 590 nm, respectively. The temperature was raised with a step of 3°C per minute from 25°C to 96°C and fluorescence readings were taken at each interval. Data was analysed as previously described [24].

### Urea-induced Equilibrium Unfolding

For equilibrium transition studies, Pim-1 wild type and variants (final concentration 40.0 µg/ml) were incubated at 10°C at increasing concentrations of urea (0−8 M) in 20 mM Tris/HCl, pH 7.5, in the presence of 0.2 M NaCl and 200 µM DTT. After 10 min, equilibrium was reached and intrinsic fluorescence emission and far-UV CD spectra (0.5-cm cuvette) were recorded in parallel at 10°C. To test the reversibility of the unfolding, Pim-1 wild type and variants were unfolded at 10°C in 7.0 M urea at 0.4 mg/ml protein concentration in 25 mM Tris/HCl, pH 7.5, in the presence of 2 mM DTT and 0.2 M NaCl. After 10 min, refolding was started by 10-fold dilution of the unfolding mixture at 10°C into solutions of the same buffer used for unfolding containing decreasing urea concentrations. The final enzyme concentration was 40 µg/ml. After 24 h, intrinsic fluorescence emission and far-UV CD spectra were recorded at 10°C.

### Thermal Denaturation Experiments

Pim-1 variants and wild type (0.20 mg/ml) were heated from 10°C to 72°C in a 0.1 cm quartz cuvette with a heating rate of 1 degree×min^−1^ controlled by a Jasco programmable Peltier element. The dichroic activity at 209 nm and the PMTV were continuously monitored in parallel every 0.5°C [21]. All the thermal scans were corrected for the solvent contribution at the different temperatures. Melting temperature (T_m_) values were calculated by taking the first derivative of the ellipticity at 209 nm with respect to temperature. All denaturation experiments were performed in triplicate.

### Data Analysis

Far-UV CD spectra recorded as a function of urea concentration were analyzed by a singular value decomposition algorithm (SVD) using the software MATLAB (Math-Works, South Natick, MA) to remove the high frequency noise and the low frequency random errors and determine the number of independent components in any given set of spectra. CD spectra in the 213–250 nm region were placed in a rectangular matrix *A* of *n* columns, one column for each spectrum collected at each time. The *A* matrix is decomposed by SVD into the product of three matrices: *A* = U*S*V ^T^, where *U* and *V* are orthogonal matrices and *S* is a diagonal matrix. The *U* matrix columns contain the basis spectra and the *V* matrix columns contain the urea dependence of each basis spectrum. Both *U* and *V* columns are arranged in terms of decreasing order of the relative weight of information, as indicated by the magnitude of the singular values in *S*. The diagonal *S* matrix contains the singular values that quantify the relative importance of each vector in *U* and *V*. The signal-to-noise ratio is very high in the earliest columns of *U* and *V* while the random noise is mainly accumulated in the latest *U* and *V* columns. The wavelength averaged spectral changes induced by increasing denaturant concentrations are represented by the columns of matrix *V*; hence, the plot of the columns of *V* versus the denaturant concentrations provides information about the observed transition. Urea-induced equilibrium unfolding was analysed by fitting baseline and transition region data to a two-state linear extrapolation model [30] according to
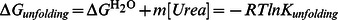
(2)where Δ*G*_unfolding_ is the free energy change for unfolding for a given denaturant concentration, Δ*G* ^H^_2_^O^ the free energy change for unfolding in the absence of denaturant and *m* a slope term which quantifies the change in Δ*G*_unfolding_ per unit concentration of denaturant, *R* the gas constant, *T* the temperature and *K*_unfolding_ the equilibrium constant for unfolding. The model expresses the signal as a function of denaturant concentration:
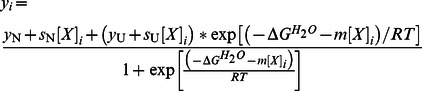
(3)where yi is the observed signal, yU and yN are the baseline intercepts for unfolded and native protein, sU and sN are the baseline slopes for the unfolded and native protein, [X]i the denaturant concentration after the ith addition, ΔGH2O the extrapolated free energy of unfolding in the absence of denaturant, m the slope of a ΔGunfolding versus [X] plot. The denaturant concentration at the midpoint of the transition, [Urea]0.5, according to equation 2, is calculated as:



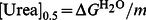
(4)The changes in intrinsic fluorescence emission spectra at increasing urea concentrations were quantified as the decrease of relative fluorescence intensity at 345 nm. The denaturation curves obtained by plotting the relative fluorescence intensities changes at 345 nm induced by increasing urea concentrations were quantitatively analyzed by two-, three- and four-state transition model. The best fit was obtained by fitting the data to a four-state transition model:
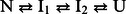
where N and U are the native and the unfolded state, respectively, and I_1_ and I_2_ are the two unfolding intermediates. The data were fitted to equation (5) which describes a four-state unfolding transition [31], [32].



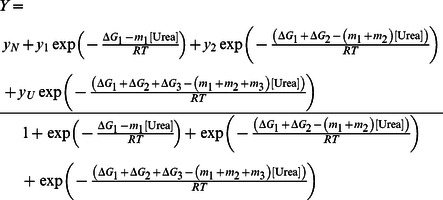
(5)The values of parameters Δ*G*_1_, Δ*G*_2_, Δ*G*_3_, *m*_1_, *m*_2_ and *m*_3_ were determined using the non-linear least fitting (GRAPHPAD Prism) to the unfolding data using equation 5. Δ*G*_1_, Δ*G*_2_ and Δ*G*_3_ represent the free energy change of each step and *m*_1_, *m*_2_ and *m*_3_ represent the free energy dependences on urea concentration ([Urea]) associated with each step. The values of *y*_N_, *y*_1_, *y*_2_ and *y*_U_ representing the signal from each species were estimated graphically and set as constant during the fit. The fractions of native, intermediate and unfolded states, *f*_N_, *f*_I1_, *f*_I2_ and *f*_U_, respectively, were calculated as described in [31], [33].

## Supporting Information

Table S1 Temperature shift data measured on Pim-1 inhibitors of the beta-carboline class and staurosporine imidazopyridazine.
(DOC)

Table S2 Temperature shift data measured on Pim-1 inhibitors of the imidazopyridazine class.
(DOC)
