# Supplementary material for: Effect of Single Amino Acid Substitution Observed in Cancer on Pim-1 Kinase Thermodynamic Stability and Structure
Source: PLoS One. 2013 Jun 5;8(6):e64824. doi: 10.1371/journal.pone.0064824 (PMC3673989; doi:10.1371/journal.pone.0064824)
Supplement: Table S1 — Temperature shift data measured on Pim-1 inhibitors of the beta-carboline class and staurosporine imidazopyridazine. (DOC) [file pone.0064824.s001.doc]

|  |  |  | **ΔT_m_ (K)** | | | | |
| --- | --- | --- | --- | --- | --- | --- | --- |
|  | Structure | **SGC ID** | **Wild type** | **Y53H** | **E124Q** | **E135K** | **E142D** |
| **CARBOILINES** |  | K01739a | 4.8 | 3.9 | 3.6 | 4.9 | 2.1 |
|  |  | K01741a | 5.1 | 3.3 | 3.3 | 5.2 | 3.3 |
|  |  | K01786a | 8.4 | 8.2 | 7.2 | 8.2 | 8.6 |
|  |  | K01844a | 9.1 | 9.0 | 7.8 | 9.0 | 9.4 |
|  |  | K01847a | 5.5 | 4.4 | 4.1 | 5.6 | 4.2 |
| STAUROSPORINE |  | K00207a | 11.7 | 10.7 | 11.7 | 11.0 | 11.3 |

**Table S3 Temperature shift data measured on Pim-1 inhibitors of the beta-carboline class and staurosporine imidazopyridazine.**
