# Supplementary material for: Effect of Single Amino Acid Substitution Observed in Cancer on Pim-1 Kinase Thermodynamic Stability and Structure
Source: PLoS One. 2013 Jun 5;8(6):e64824. doi: 10.1371/journal.pone.0064824 (PMC3673989; doi:10.1371/journal.pone.0064824)
Supplement: Table S2 — Temperature shift data measured on Pim-1 inhibitors of the imidazopyridazine class. (DOC) [file pone.0064824.s002.doc]

**Table S4. Temperature shift data measured on Pim-1 inhibitors of the imidazopyridazine class.**

|  |  |  | **ΔT_m_ (K)** | | | | |
| --- | --- | --- | --- | --- | --- | --- | --- |
|  | Structure | **SGC ID** | **Wild type** | **Y53H** | **E124Q** | **E135K** | **E142D** |
| **IMIDAZOPYRIDAZINES** |  | K00478 | 6.0 | 4.5 | 4.9 | 5.9 | 4.8 |
|  |  | K00486 | 9.3 | 8.3 | 9.5 | 9.9 | 9.0 |
|  |  | K00487 | 7.7 | 7.0 | 7.6 | 7.5 | 8.1 |
|  |  | K00488 | 6.3 | 6.0 | 6.0 | 6.7 | 6.9 |
|  |  | K00499 | 5.5 | 5.9 | 5.6 | 6.0 | 6.6 |
|  |  | K00500 | 8.5 | 7.8 | 8.4 | 8.3 | 8.5 |
|  |  | K00512 | 8.4 | 8.1 | 8.5 | 8.9 | 8.8 |
|  |  | K00514 | 6.1 | 5.6 | 6.1 | 7.5 | 6.2 |
|  |  | K00516 | 8.2 | 6.9 | 8.2 | 9.0 | 7.7 |
|  |  | K00518 | 5.6 | 4.6 | 4.3 | 6.4 | 5.3 |
|  |  | K00520 | 6.8 | 5.5 | 5.0 | 6.5 | 6.4 |
